# HAIC combined with atezolizumab and bevacizumab versus lenvatinib in advanced hepatocellular carcinoma: a multicenter propensity score matching analysis

**DOI:** 10.3389/fonc.2026.1880527

**Published:** 2026-08-24

**Authors:** Lei Zhao, Dan Liu, Chao Ma, Yue Hu, Shu Liu

**Affiliations:** 1 Department of Medical Oncology, The Affiliated Yantai Yuhuangding Hospital of Qingdao University, Yantai, China; 2 Surgical Anesthesia Center, The Seventh Affiliated Hospital Sun Yat-sen University, Shenzhen, China; 3 Department of Interventional Radiology, The Third Affiliated Hospital of Sun Yat-sen University, Guangzhou, China

**Keywords:** atezolizumab, bevacizumab, hepatic arterial infusion chemotherapy, hepatocellular carcinoma, lenvatinib

## Abstract

**Purpose:**

This study aimed to evaluate the clinical efficacy and safety of hepatic arterial infusion chemotherapy (HAIC) combined with atezolizumab and bevacizumab (HTA) versus HAIC combined with lenvatinib (HL) in the treatment of advanced hepatocellular carcinoma (HCC).

**Methods:**

A multicenter retrospective study was conducted between June 2021 and June 2025, reviewing advanced HCC patients treated with either HTA or HL. To mitigate initial differences between the two groups, propensity score matching (PSM) was applied. Tumor response was assessed using the RECIST version 1.1 criteria. The primary endpoint was overall survival (OS). Secondary efficacy outcomes included progression-free survival (PFS), objective response rate (ORR), and disease control rate (DCR). Safety was assessed by the incidence and severity of adverse events (AEs).

**Results:**

A total of 187 patients received HTA, and 119 patients received HL. Following PSM, 106 patients were assigned to each group. The HTA group demonstrated a significantly longer median OS (25.8 months vs. 16.4 months; hazard ratio [HR], 0.62, *P* = 0.012) compared to the HL group. However, no significant difference in median PFS was observed (9.7 months vs. 7.6 months; HR, 0.88, *P* = 0.407). Additionally, the two groups showed comparable ORR (56.6% vs. 53.8%, *P* = 0.78) and DCR (86.8% vs. 84.9%, *P* = 0.84). There were no significant differences in the incidence of all AEs or grade 3/4 AEs between the two groups. Importantly, all AEs were manageable and acceptable, with no grade 5 AEs reported in either group.

**Conclusion:**

HTA significantly improved overall survival compared to HL in patients with advanced HCC, with manageable safety profiles.

## Introduction

Hepatocellular carcinoma (HCC) is the sixth most prevalent cancer worldwide and the third leading cause of cancer-related death (1). Its highly asymptomatic nature results in over 60% of cases advancing to an inoperable stage by the time of diagnosis in China, limiting treatment options and leading to poor prognosis (2). Without treatment, the median overall survival (OS) for these patients is alarmingly short, ranging from 2.7 to 4.0 months (3). Thus, there is an urgent need for effective therapies that can substantially improve survival outcomes.

The Barcelona Clinic Liver Cancer (BCLC) staging system recommends systemic therapies, including targeted therapies and immunotherapy, for patients with advanced HCC at BCLC stage C (4). Sorafenib, the first-line treatment since 2008, provides limited benefit, with an OS of only 5.5 months for patients with portal vein tumor thrombosis (PVTT) (5). In 2018, lenvatinib emerged as a viable alternative, demonstrating superior overall response rate (ORR) (29.6% vs. 6.9%; p < 0.001), progression-free survival (PFS) (7.2 months vs. 4.6 months; p < 0.001), and non-inferior OS (13.6 months vs. 12.3 months) compared to sorafenib in the REFLECT study (6). More recently, immune checkpoint inhibitors (ICIs) have shown promise for advanced HCC and based on the favorable outcomes of the IMbrave150 trial, atezolizumab-bevacizumab combination therapy is now recommended as first-line treatment (7). However, multi-tyrosine kinase inhibitors (TKIs) like lenvatinib and sorafenib remain alternative options when combination therapy is not feasible.

In addition to systemic therapies, hepatic arterial infusion chemotherapy (HAIC) using FOLFOX-based regimens has demonstrated superior outcomes over sorafenib alone for advanced HCC (8). By directly delivering concentrated chemotherapy to the liver, HAIC offers significant local antitumor effects and is widely used for both primary and metastatic hepatic malignancies. Both Chinese and Japanese guidelines recommend HAIC, particularly for advanced HCC with major PVTT (9, 10). A phase III trial has confirmed survival benefits from combining sorafenib with HAIC using a modified FOLFOX regimen (11). Recent retrospective studies further suggest that combining HAIC with lenvatinib leads to significant improvements in PFS, OS, and ORR compared to lenvatinib monotherapy (12). Moreover, many studies have also shown that HAIC combined with TA (HTA) offered survival benefits in advanced HCC (13). However, the comparative outcomes of HL versus HTA have yet to be fully explored.

This retrospective study aims to evaluate the efficacy of HTA versus HL in patients with advanced HCC.

## Methods

### Study design and patients

This retrospective study analyzed patients with advanced HCC who underwent HAIC in combination with either lenvatinib (HL) or atezolizumab plus bevacizumab (HTA) between June 2021 and June 2025 at the Affiliated Yantai Yuhuangding Hospital of Qingdao University and the Third Affiliated Hospital of Sun Yat-sen University in China. Patients were categorized into two cohorts: the HL group and the HTA group.

All participants underwent pre-treatment assessments including magnetic resonance imaging (MRI), dynamic abdominal computed tomography (CT), and/or abdominal ultrasonography. Eligibility criteria included: (1) aged 18–75 years; (2) HCC confirmed radiologically or pathologically according to the American Association for the Study of Liver Diseases (AASLD) guidelines; (3) Barcelona Clinic Liver Cancer (BCLC) stage C; (4) no prior anti-tumor therapy within six months; (5) preserved liver function (Child-Pugh class A or B); (6) Eastern Cooperative Oncology Group (ECOG) performance status ≤ 1; (7) absence of other malignancies within the preceding five years; (8) adequate organ and hematologic function to receive HAIC-based combination therapy; and (9) an anticipated survival of more than 3 months at treatment initiation. Patients were considered eligible if HAIC-based combination therapy was planned at treatment initiation and they initiated treatment with either HTA or HL. In routine clinical practice, HAIC was administered in repeated cycles, and tumor response was generally assessed after every two HAIC cycles. Exclusion criteria were: (1) history of esophageal variceal bleeding within 3 months; (2) heart failure or concomitant ischemic heart disease; (3) impaired organ or hematologic function; (4) inconsistent treatment; and (5) incomplete follow-up data.

Laboratory tests and imaging studies were conducted within one week prior to the initiation of therapy. The study protocol was approved by the Ethics Committee of the Affiliated Yantai Yuhuangding Hospital of Qingdao University, and the requirement for informed consent was waived due to the retrospective design.

### Treatment procedures

#### HAIC protocol

HAIC was administered following established protocols (8). Catheter placement was tailored according to tumor size, location, and arterial supply, with the tip positioned in tumor-feeding hepatic artery branches. The HAIC regimen consisted of oxaliplatin (130 mg/m², infused over 2 hours on day 1), leucovorin (200 mg/m², infused over 2 hours on day 1), and fluorouracil (400 mg/m² bolus over 15 minutes, followed by 2400 mg/m² continuous infusion over 46 hours on days 1–2). Catheters and sheaths were removed upon completion of each session. Subsequent HAIC cycles were scheduled every three weeks based on operator evaluation, with a maximum of eight cycles.

#### Lenvatinib administration

Lenvatinib was initiated 3–5 days after the first HAIC session at a daily dose of 12 mg for patients ≥60 kg and 8 mg for those <60 kg. For grade 1–2 adverse events (AEs), the dose was maintained and supportive care provided. In the event of grade 3–4 AEs, doses were reduced to 8 mg or 4 mg, respectively, or administered every other day until resolution. Persistent severe AEs prompted temporary discontinuation until improvement.

#### Atezolizumab plus bevacizumab (TA) administration

Patients received intravenous atezolizumab (1200 mg) plus bevacizumab (15 mg/kg) 1–7 days post-HAIC every three weeks. Grade 1–2 AEs did not require dose modification, whereas grade 3–4 AEs necessitated treatment interruption until resolution. Recurrent severe AEs upon re-administration warranted permanent discontinuation of the TA regimen.

#### Conversion surgery

Decisions regarding conversion to salvage liver resection were made by a multidisciplinary team (MDT), with procedures performed by surgeons experienced in hepatic resection.

### Follow-up and assessment

Tumor response was evaluated by dynamic enhanced CT or MRI after two HAIC sessions. Follow-up assessments were conducted by the same MDT. Tumor responses were categorized according to RECIST v1.1 as complete response (CR), partial response (PR), stable disease (SD), or progressive disease (PD). Objective response rate (ORR) and disease control rate (DCR) were defined as CR/PR and CR/PR/SD, respectively. Progression-free survival (PFS) was defined as the interval from admission to disease progression or death from any cause. Overall survival (OS) was calculated from the initiation of HAIC to death or last follow-up. Adverse events were graded according to CTCAE v5.0.

### Propensity score matching

Propensity score matching (PSM) was performed to minimize selection bias. Propensity scores were estimated using a multivariable logistic regression model that included prespecified baseline characteristics considered clinically relevant to treatment selection and prognosis, including age, sex, ECOG performance status, etiology, ALBI score, Child-Pugh class, tumor size, tumor number, AFP level, portal vein tumor thrombosis (PVTT), and extrahepatic metastasis. Variable selection was based on clinical relevance rather than statistical significance alone. No interaction terms or nonlinear transformations were included in the propensity score model. One-to-one nearest-neighbor matching without replacement was performed using a caliper width of 0.2 standard deviations of the logit of the propensity score. Covariate balance after matching was evaluated using standardized mean differences (SMDs), with values <0.1 indicating adequate balance.

### Statistical analysis

The analytic dataset was reviewed for missingness before statistical analysis. No missing data were identified for baseline covariates included in the propensity score model, treatment assignment, survival outcomes, tumor response assessment, or adverse event reporting; therefore, no imputation was performed. Statistical analyses were conducted using R (v4.0.3) and SPSS (v27.0). Continuous variables were expressed as mean ± SD or median (IQR) and compared using Student’s *t*-test or Mann–Whitney *U* test, as appropriate. Categorical variables were presented as counts and percentages and analyzed with *χ²* or Fisher’s exact tests. Time-to-event outcomes were estimated using the Kaplan–Meier method, with differences evaluated via log-rank tests. Univariable and multivariable Cox regression analyses were performed to identify prognostic factors. Variables with *P* < 0.1 in univariable analysis were included in multivariable models. Statistical significance was set at two-sided *P* < 0.05.

## Results

### Patient characteristics

**Figure 1:**
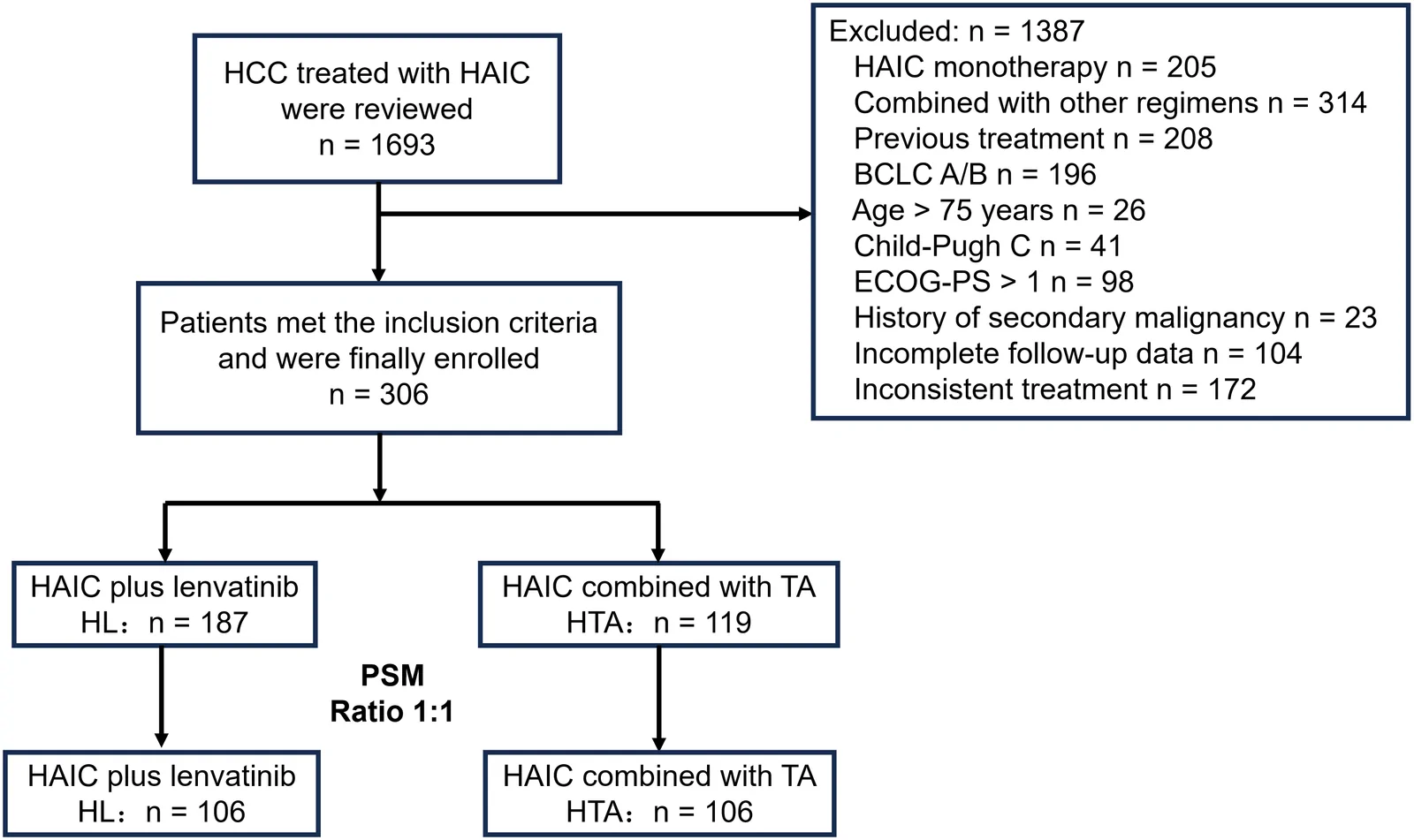
Patient flowchart. PSM, propensity score matching; HL, HAIC plus lenvatinib; HAIC, hepatic arterial infusion chemotherapy; HTA, HAIC combined with bevacizumab and atezolizumab; ECOG, Eastern Cooperative Oncology Group; PS, performance score.

The patient selection process is summarized in **Figure 1**. Of 306 patients with advanced HCC assessed for eligibility, 187 received HAIC plus lenvatinib (HL group), and 119 received HAIC combined with atezolizumab and bevacizumab (HTA group), with median follow-up durations of 49.8 and 52.3 months, respectively. Significant differences in age, ECOG performance status, intrahepatic tumor number, and AFP levels were noted between the groups. To address potential selection bias, propensity score matching (PSM) yielded 106 patients in each cohort, with balanced baseline characteristics (all *P* > 0.2) (**Table 1**). Covariate balance was assessed using standardized mean differences (SMDs), with an absolute SMD < 0.1 considered indicative of adequate balance. Detailed balance diagnostics are presented in **Supplementary Table 1**.

**Table 1:** Baseline characteristics of the study patients before and after PSM.

Characteristics	Before matching			After matching		
	HL (n = 187)	HTA (n = 119)	P	HL (n = 106)	HTA (n = 106)	P
Age (Mean ± SD, year)	52 ± 12	48 ± 12	<0.01	50 ± 12	49 ± 12	0.40
< 50	76 (40.6%)	60 (50.4%)	0.09	48 (45.3%)	53 (50.0%)	0.49
≥ 50	111 (59.4%)	59 (49.6%)		58 (54.7%)	53 (50.0%)	
Sex			0.21			0.80
Female	14 (7.5%)	14 (11.8%)		8 (7.5%)	9 (8.5%)	
Male	173 (92.5%)	105 (88.2%)		98 (92.5%)	97 (91.5%)	
ECOG-PS			<0.01			0.42
0	140 (74.9%)	107 (89.9%)		90 (84.9%)	94 (88.7%)	
1	47 (25.1%)	12 (10.1%)		16 (15.1%)	12 (11.3%)	
Etiology			0.52			>0.99
No-hepatitis	12 (6.4%)	11 (9.2%)		6 (5.7%)	6 (5.7%)	
HBV	174 (93.0%)	107 (89.9%)		99 (93.4%)	99 (93.4%)	
HCV	1 (0.5%)	1 (0.8%)		1 (0.9%)	1 (0.9%)	
Child-Pugh			>0.99			0.84
A	165 (88.2%)	105 (88.2%)		93 (87.7%)	92 (86.8%)	
B	22 (11.8%)	14 (11.8%)		13 (12.3%)	14 (13.2%)	
ALBI			0.66			0.88
1	67 (35.8%)	49 (41.2%)		46 (43.4%)	42 (39.6%)	
2	116 (62.0%)	68 (57.1%)		58 (54.7%)	62 (58.5%)	
3	4 (2.1%)	2 (1.7%)		2 (1.9%)	2 (1.9%)	
Size (Mean ± SD, cm)	12.0 ± 3.3	12.5 ± 3.4	0.22	12.3 ± 3.7	12.1 ± 3.2	0.62
< 10	52 (27.8%)	33 (27.7%)	0.99	31 (29.2%)	30 (28.3%)	0.88
≥ 10	135 (72.2%)	86 (72.3%)		75 (70.8%)	76 (71.7%)	
Number			<0.01			>0.99
Single	77 (41.2%)	26 (21.8%)		24 (22.6%)	24 (22.6%)	
Multiple	110 (58.8%)	93 (78.2%)		82 (77.4%)	82 (77.4%)	
AFP (ug/L)			0.04			0.89
< 400	56 (29.9%)	49 (41.2%)		41 (38.7%)	40 (37.7%)	
≥ 400	131 (70.1%)	70 (58.8%)		65 (61.3%)	66 (62.3%)	
PVTT			0.96			0.85
Absent	35 (18.7%)	22 (18.5%)		18 (17.0%)	17 (16.0%)	
Present	152 (81.3%)	97 (81.5%)		88 (83.0%)	89 (84.0%)	
Extrahepatic metastasis			0.76			0.78
Absent	85 (45.5%)	52 (43.7%)		48 (45.3%)	46 (43.4%)	
Present	102 (54.5%)	67 (56.3%)		58 (54.7%)	60 (56.6%)	

Values are presented as n (%).

*P* values were calculated using a two-sided χ^2^ test.

PSM, propensity score matching; HL, HAIC plus lenvatinib; HAIC, hepatic arterial infusion chemotherapy; HTA, HAIC combined with bevacizumab and atezolizumab; ECOG, Eastern Cooperative Oncology Group; PS, performance score; HBV, hepatitis B virus; HCV, hepatitis C virus; ALBI, albumin-bilirubin; AFP, alpha-fetoprotein; PVTT, portal vein tumor thrombus.

Most patients had HBV-related HCC with preserved liver function. The median tumor diameter exceeded 10 cm in both groups, and the majority had multiple intrahepatic lesions before and after PSM, reflecting a substantial tumor burden. Before PSM, the median number of HAIC sessions was 5 (range 2–8) in the HL group and 4 (range 2–8) in the HTA group.

### Tumor response

Best tumor responses according to RECIST v1.1 are presented in **Table 2**. The HTA group demonstrated comparable ORR (before PSM: 50.8% vs. 53.8%, *P* = 0.64; after PSM: 53.8% vs. 56.6%, *P* = 0.78) and DCR (before PSM: 83.4% vs. 84.9%, *P* = 0.87; after PSM: 84.9% vs. 86.8%, *P* = 0.84) relative to the HL group.

**Table 2:** Treatment efficacy evaluated by RECIST 1.1 criteria before and after PSM.

	Before matching			After matching		
	HL (n = 187)	HTA (n = 119)	*P*	HL (n = 106)	HTA (n = 106)	*P*
CR	2 (1.1%)	3 (2.5%)		1 (1.8%)	2 (1.9%)	
PR	93 (49.7%)	61 (51.3%)		56 (52.8%)	58 (54.7%)	
SD	61 (32.6%)	37 (31.1%)		33 (31.1%)	32 (30.2%)	
PD	31 (16.6%)	18 (15.1%)		16 (15.1%)	14 (13.2%)	
ORR	95 (50.8%)	64 (53.8%)	0.64	57 (53.8%)	60 (56.6%)	0.78
DCR	156 (83.4%)	101 (84.9%)	0.87	90 (84.9%)	92 (86.8%)	0.84

Summary of best response.

Values are presented as n (%).

*P* values were calculated using a two-sided χ^2^ test.

PSM, propensity score matching; RECIST, response evaluation criteria in solid tumors; HL, HAIC plus lenvatinib; HAIC, hepatic arterial infusion chemotherapy; HTA, HAIC combined with bevacizumab and atezolizumab; CR, complete response; PR, partial response; SD, stable disease; PD, progressive disease; ORR, objective response rate; DCR, disease control disease.

**Figure 2:**
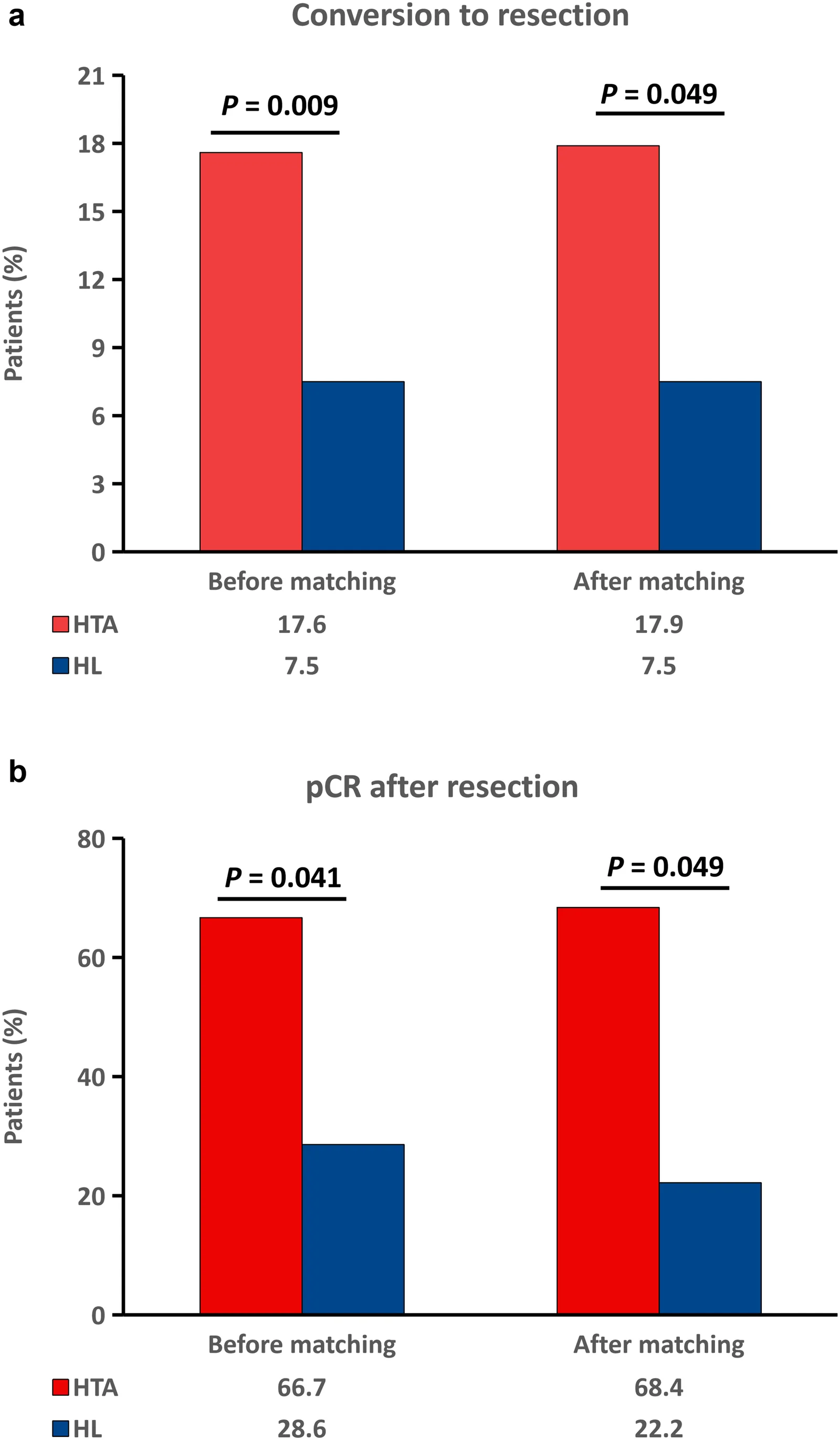
The rate of conversion to resection **(a)** and pCR after resection **(b)** between the two groups before and after PSM. *P* values were calculated using a two-sided *χ^2^* test. PSM, propensity score matching; HL, HAIC plus lenvatinib; HAIC, hepatic arterial infusion chemotherapy; HTA, HAIC combined with bevacizumab and atezolizumab.

**Figure 2** depicts patients who underwent surgical resection following tumor downstaging or substantial shrinkage. Prior to PSM, a higher proportion of patients in the HTA group received resection compared with patients in the HL group [17.6% (21/119) vs. 7.5% (14/187); *P* = 0.009], with a greater rate of pathological complete response (pCR) (66.7% vs. 28.6%; *P* = 0.041). After PSM, resection remained more frequent in the HTA group [17.9% (19/106) vs. 7.5% (8/106); *P* = 0.038], with a higher pCR rate (68.4% vs. 25.0%; *P* = 0.049).

### Survival outcomes

**Figure 3:**
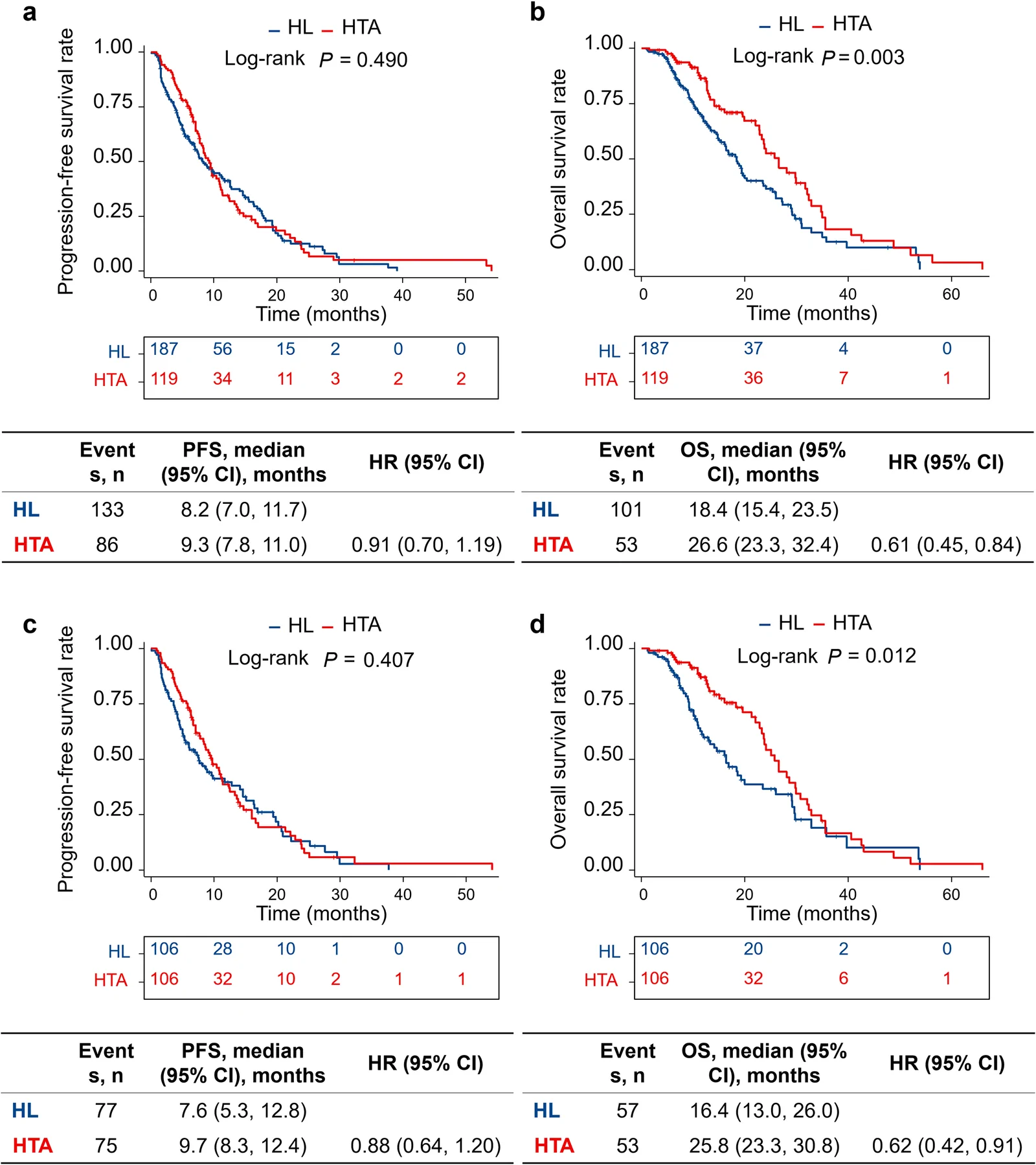
Kaplan-Meier curves comparing OS and PFS among patients who underwent HTA versus HL before **(a, b)** and after **(c, d)** PSM. *P* values were calculated using Log-rank test. PSM, propensity score matching; HL, HAIC plus lenvatinib; HAIC, hepatic arterial infusion chemotherapy; HTA, HAIC combined with bevacizumab and atezolizumab; OS, overall survival; PFS, progression-free survival; HR: hazard ratio; CI, confidence interval.

Before PSM, 101 patients in the HL group and 53 patients in the HTA group had died by the end of the follow-up. Median OS was significantly longer in the HTA group (26.6 months, 95% CI: 23.3–32.4) than in the HL group (18.4 months, 95% CI: 15.5–23.5; HR: 0.61, 95% CI: 0.45–0.84; *P* = 0.003), whereas median PFS was similar (9.3 months, 95% CI: 7.8–11.0 vs. 8.2 months, 95% CI: 7.0–11.7; HR: 0.91, 95% CI: 0.70–1.19; *P* = 0.490). Among the 106 PSM-matched pairs, the HTA group maintained longer OS (25.8 months, 95% CI: 23.3–30.8 vs. 16.4 months, 95% CI: 13.0–26.0; HR: 0.62, 95% CI: 0.42–0.91; *P* = 0.012), while PFS differences were not statistically significant (9.7 months, 95% CI: 8.3–12.4 vs. 7.6 months, 95% CI: 5.3–12.8; HR: 0.88, 95% CI: 0.64–1.20; *P* = 0.407) (**Figure 3**).

To evaluate whether the overall survival benefit was primarily driven by subsequent conversion surgery, a sensitivity analysis was performed after excluding patients who underwent conversion resection. After exclusion, the HTA group continued to demonstrate a significant OS advantage compared with the HL group before PSM (26.6 vs. 18.2 months, HR: 0.55, 95% CI: 0.39–0.76; *P* < 0.001) and after PSM (26.6 vs. 16.4 months; HR: 0.55, 95% CI: 0.37–0.82; *P* = 0.003) (**Supplementary Figure 1**), indicating that the observed survival benefit was not solely attributable to conversion surgery.

### Univariate and multivariate analysis

Results of univariate and multivariate Cox regression analyses for OS and PFS are summarized in **Table 3**. Multivariate analysis identified HAIC plus lenvatinib as an independent risk factor for OS (HR: 0.40; 95% CI: 0.26–0.62; *P* < 0.001). Additionally, ECOG-PS 1, Child-Pugh class B, and metastasis independently predicted shorter PFS, while Child-Pugh class B was an independent predictor of OS.

**Table 3:** Univariate and multivariate analyses of predictors of survival after treatment.

Characteristics	Overall survival				Progression-free survival			
	Univariate analysis		Multivariate analysis		Univariate analysis		Multivariate analysis	
	HR (95% CI)	*P*	HR (95% CI)	*P*	HR (95% CI)	*P*	HR (95% CI)	*P*
Treatment (HL)	0.40 (0.26 - 0.61)	<0.001	0.40 (0.26 - 0.62)	<0.001	0.89 (0.66 - 1.19)	0.43		
Age (< 50y)	0.93 (0.64 - 1.34)	0.69			0.86 (0.65 - 1.14)	0.30		
Sex (Female)	0.75 (0.44 - 1.28)	0.29			0.80 (0.51 - 1.26)	0.33		
ECOG-PS (0)	1.12 (0.67 - 1.85)	0.67			0.68 (0.45 - 1.03)	0.07	0.65 (0.43 - 0.99)	0.04
Etiology (No-hepatitis)	1.01 (0.49 - 2.07)	0.99			0.68 (0.41 - 1.14)	0.14		
ALBI (1)								
2	1.15 (0.78 - 1.69)	0.47	1.09 (0.74 - 1.62)	0.66	0.90 (0.67 - 1.20)	0.46		
3	2.70 (0.83 - 8.78)	0.10	1.17 (0.30 - 4.51)	0.82	1.11 (0.41 - 3.05)	0.83		
Child-Pugh (A)	1.97 (1.10 - 3.54)	0.02	1.81(0.93 - 3.54)	0.08	1.51 (0.98 - 2.32)	0.06	1.61 (1.05 - 2.49)	0.03
Size (< 10cm)	0.94 (0.63 - 1.40)	0.76			1.11 (0.80 - 1.52)	0.54		
Number (Single)	1.11 (0.76 - 1.64)	0.58			1.10 (0.82 - 1.48)	0.54		
AFP (< 400μg/L)	1.38 (0.92 - 2.07)	0.12			1.25 (0.92 - 1.69)	0.15		
PVTT (Absent)	1.36 (0.85 - 2.20)	0.20			0.89 (0.63 - 1.26)	0.50		
Metastasis (Absent)	1.09 (0.75 - 1.59)	0.64			1.42 (1.07 - 1.90)	0.02	1.45 (1.09 - 1.94)	0.01

Univariable and multivariable Cox regression analyses were performed to identify the factors associated with survival. Factors with *P* < 0.1 in univariate analysis were included in multivariate analysis. Two-sided P<0.05 was defined as statistically significant.

HL, HAIC plus lenvatinib; HAIC, hepatic arterial infusion chemotherapy; HTA, HAIC combined with bevacizumab and atezolizumab; ECOG, Eastern Cooperative Oncology Group; PS, performance score; HBV, hepatitis B virus; HCV, hepatitis C virus; ALBI, albumin-bilirubin; AFP, alpha-fetoprotein; PVTT, portal vein tumor thrombus.

The category shown in parentheses was the reference category.

### Subgroup analysis

**Figure 4:**
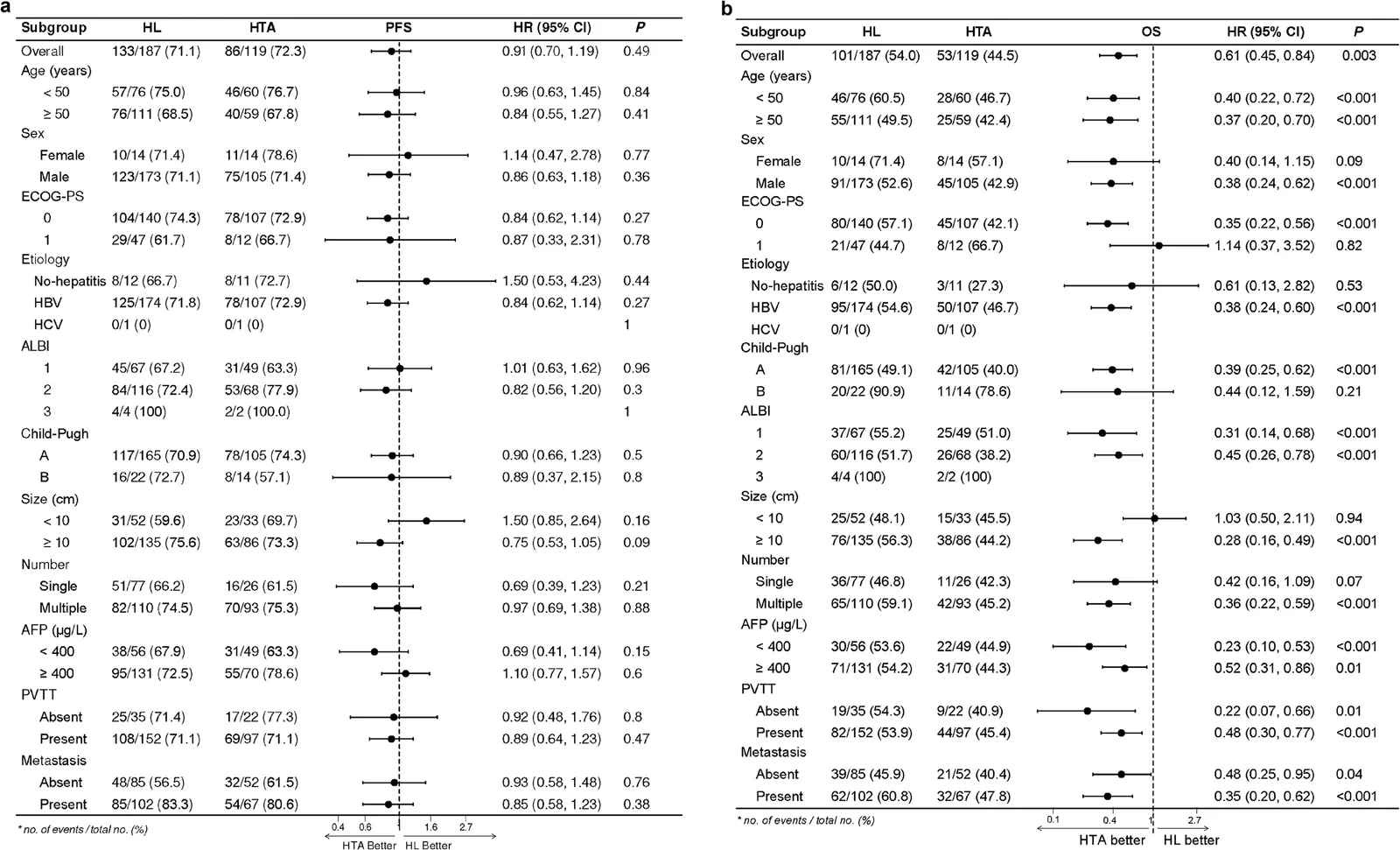
Forest plots based on OS **(a)** and PFS **(b)** of each subgroup. *P* values were calculated using Log-rank test. PSM, propensity score matching; HL, HAIC plus lenvatinib; HAIC, hepatic arterial infusion chemotherapy; HTA, HAIC combined with bevacizumab and atezolizumab; ECOG, Eastern Cooperative Oncology Group; PS, performance score; HBV, hepatitis B virus; ALBI, albumin-bilirubin; AFP, alpha-fetoprotein; PVTT, portal vein tumor thrombus; OS, overall survival; PFS, progression-free survival; HR: hazard ratio; CI: confidence interval.

Forest plots (**Figure 4**) demonstrate treatment effects across subgroups. The HTA group consistently exhibited superior OS across nearly all subgroups, whereas PFS differences between groups were not statistically significant, indicating the broad efficacy of HTA in advanced HCC.

### Safety

Treatment-related adverse events (TRAEs) are summarized in **Table 4**. The most frequent TRAEs were nausea, diarrhea, and abdominal pain, predominantly grade 1–2. Overall incidences of any-grade or grade 3–4 TRAEs were comparable between groups. Tyrosine kinase inhibitor–related AEs, including hand–foot skin reactions and rash, were more common in the HL group, while gastrointestinal hemorrhage rates did not differ significantly. All AEs were manageable, and no treatment-related deaths occurred.

**Table 4:** Treatment-related adverse events.

Adverse event	Any grade			Grade 3/4		
	HL (n = 187)	HTA (n = 119)	*P*	HL (n = 187)	HTA (n = 119)	*P*
Overall incidence	139 (74.3)	92 (77.3)	0.588	51 (27.3)	35 (29.4)	0.697
Abdominal pain	58 (31.0)	40 (33.6)	0.706	21 (11.2)	15 (12.6)	0.719
Nausea	88 (47.1)	59 (49.6)	0.725	34 (18.1)	22 (18.5)	0.999
Diarrhea	62 (33.2)	47 (39.5)	0.272	23 (12.3)	18 (15.1)	0.495
Fever	28 (15.0)	15 (12.6)	0.616	9 (4.8)	5 (4.2)	0.999
Decreased appetite	71 (38.0)	50 (42.0)	0.549	12 (6.4)	7 (5.9)	0.999
Rash	41 (21.9)	6 (5.0)	<0.001	9 (4.8)	0 (0)	0.014
Fatigue	36 (19.3)	23 (19.3)	0.999	15 (8.0)	8 (6.7)	0.825
Hypoproteinemia	35 (18.7)	28 (23.5)	0.314	19 (10.2)	11 (9.2)	0.846
Elevated bilirubin	49 (26.2)	32 (26.9)	0.895	17 (9.1)	9 (7.6)	0.681
Hypothyroidism	17 (9.1)	2 (1.7)	0.007	2 (1.1)	0 (0)	0.523
Neurologic toxicity	52 (27.8)	29 (24.4)	0.595	3 (1.6)	1 (0.8)	0.999
Leukopenia	40 (21.4)	26 (21.8)	0.999	7 (3.7)	5 (4.2)	0.999
Thrombocytopenia	35 (18.7)	27 (22.7)	0.466	12 (6.4)	6 (5.0)	0.804
Hypertension	47 (25.1)	19 (16.0)	0.064	9 (4.8)	3 (2.5)	0.380
Hand-foot skin reaction	29 (15.5)	0 (0)	<0.001	8 (4.3)	0 (0)	0.025
Dysphonia	23 (12.3)	2 (1.7)	<0.001	0 (0)	0 (0)	0.999
Proteinuria	38 (32.5)	27 (22.7)	0.668	14 (7.5)	8 (6.7)	0.999
Bleeding (gingiva)	18 (9.6)	11 (9.2)	0.999	0 (0)	0 (0)	0.999
Bleeding (gastrointestinal)	5 (2.7)	4 (3.4)	0.739	5 (2.7)	4 (3.4)	0.739
Immune-related AEs	0 (0)	11 (9.2)	<0.001	0 (0)	3 (2.5)	0.058

Values are presented as n (%).

P values were calculated using a two-sided *χ2* test.

HL, HAIC plus lenvatinib; HAIC, hepatic arterial infusion chemotherapy; HTA, HAIC combined with bevacizumab and atezolizumab.

## Discussion

In this multicenter retrospective study, we demonstrated that the combination of HAIC with atezolizumab plus bevacizumab (HTA) was associated with a clinically meaningful improvement in overall survival (OS), extending survival by 9.4 months compared with HAIC plus lenvatinib (HL) in patients with advanced hepatocellular carcinoma (HCC). Notably, this survival advantage was observed despite comparable progression-free survival (PFS) and objective response rates (ORR) between the two groups, and the consistency of these findings across all prespecified subgroups further supports the robustness of our results. In addition, a higher proportion of patients in the HTA group underwent salvage liver resection following tumor regression or necrosis, with more patients achieving pathological complete response (pCR), suggesting that HTA may induce deeper or more biologically meaningful tumor responses. Importantly, both treatment regimens demonstrated manageable and comparable safety profiles. Collectively, these findings indicate that HTA may confer superior long-term survival benefit compared with HL in advanced HCC.

The role of HAIC in advanced HCC remains regionally heterogeneous. While most international guidelines have not incorporated HAIC as a standard first-line therapy, it has been widely adopted in China and other parts of Asia. This discrepancy is likely multifactorial. Patients with HCC in Chinese populations often present with a higher intrahepatic tumor burden, as also reflected in our cohort, rendering systemic therapy alone insufficient for rapid disease control (9). In this context, locoregional approaches such as HAIC can achieve prompt tumor debulking, thereby enhancing overall treatment efficacy. Indeed, multiple prior studies have shown that FOLFOX-based HAIC combined with systemic therapy significantly improves response rates and survival outcomes compared with systemic therapy alone. In addition, differences in healthcare infrastructure and patient acceptance contribute to this divergence. HAIC, as an invasive procedure, may be less feasible in Western settings, whereas in China, well-established interventional oncology systems allow for timely and repeated administration (14). As a result, HAIC-based combination strategies have become a cornerstone of real-world treatment paradigms for advanced HCC in this region.

Traditionally, HAIC has been regarded as a locoregional therapy with limited impact on extrahepatic disease. However, accumulating evidence suggests a broader antitumor effect. Chemotherapeutic agents delivered via hepatic arterial infusion may enter the systemic circulation and exert activity beyond the liver. More importantly, HAIC may act synergistically with systemic therapies (15). Locoregional tumor destruction can induce immunogenic cell death, increase tumor antigen release, and promote dendritic cell activation, thereby enhancing T-cell priming (16). Concurrently, anti-VEGF therapy (bevacizumab) may normalize tumor vasculature, reduce hypoxia, and alleviate immunosuppressive signaling within the tumor microenvironment, facilitating immune cell infiltration. The addition of immune checkpoint blockade further amplifies this effect by restoring antitumor immunity (17). Together, these mechanisms may transform an immunologically “cold” tumor into a more responsive “hot” phenotype, providing a strong biological rationale for the observed synergy between HAIC and immunotherapy-based regimens.

To date, no prospective randomized trials have directly compared atezolizumab plus bevacizumab with lenvatinib in the context of HAIC-based therapy. Existing retrospective studies have reported inconsistent results, with some suggesting superiority of immunotherapy-based combinations and others demonstrating comparable efficacy (18). Our findings provide additional insight by showing that, when combined with HAIC, the HTA regimen yields a significant OS benefit without improvement in PFS or ORR. One plausible explanation is that both regimens achieve similar early tumor control, as reflected by comparable response rates and disease stabilization. However, immune checkpoint inhibitors are characterized by delayed but durable responses, often resulting in a “long-tail” survival effect (19). When combined with HAIC-induced tumor modulation, this effect may be further enhanced, leading to prolonged survival despite similar early efficacy endpoints.

The discordance between PFS and OS observed in this study warrants careful interpretation. Although PFS is commonly used as a surrogate endpoint, its ability to fully capture the clinical benefit of immunotherapy-based treatment strategies remains imperfect (20). Similar discrepancies between PFS and OS have been reported in landmark HCC trials, including IMbrave150, where the magnitude of survival benefit exceeded that predicted by PFS alone. Several factors may contribute to this phenomenon. First, atypical response patterns associated with immunotherapy, such as pseudoprogression and delayed responses, may not be adequately reflected by conventional RECIST-based assessments (21). Second, HAIC-induced tumor necrosis and treatment-related radiologic changes may complicate response evaluation and potentially affect the accuracy of progression assessment (22). Third, post-progression management, including subsequent systemic therapies and conversion treatments, may influence long-term survival outcomes. Given the retrospective nature of this study, the impact of subsequent therapies could not be fully accounted for and therefore may have contributed to the observed OS difference. Importantly, from a real-world perspective, overall survival reflects the cumulative effectiveness of the entire treatment strategy rather than the isolated effect of first-line therapy alone (23). Consequently, while the observed OS benefit associated with HTA is clinically meaningful, it should be interpreted cautiously and viewed as hypothesis-generating evidence that warrants prospective validation in studies with standardized treatment sequencing.

From an external perspective, our findings should be interpreted with caution. The majority of patients in this study had preserved liver function (Child-Pugh A) and were treated in experienced centers, which may limit generalizability. Furthermore, etiological differences in HCC—such as the predominance of hepatitis B virus infection in Asian populations—may influence tumor biology and treatment response (24). Validation in broader and more diverse populations is therefore warranted. Besides, because formal tests for interaction were not performed and several subgroup sample sizes were relatively small, the subgroup findings should be interpreted as exploratory and hypothesis-generating.

The optimal integration and sequencing of HAIC with systemic therapy also remain to be defined. In this study, HAIC was administered concurrently with systemic treatment; however, alternative approaches may offer additional benefit. For example, induction HAIC may reduce tumor burden and enhance subsequent immunotherapy efficacy, whereas early systemic therapy may improve vascular normalization and drug delivery (25, 26). These hypotheses are supported by emerging evidence but require validation in prospective trials.

In terms of safety, the HTA regimen demonstrated a tolerable profile comparable to that of HL. Concerns regarding gastrointestinal bleeding and immune-related adverse events with atezolizumab plus bevacizumab were not associated with increased toxicity in this study. Similarly, the incidence of grade 3/4 adverse events was comparable between groups. These findings are clinically relevant, as they indicate that the survival benefit of HTA is achieved without compromising safety, supporting its feasibility in routine practice.

Several limitations should be acknowledged. First, the retrospective design introduces potential selection bias, although propensity score matching was applied to mitigate confounding. Second, the follow-up duration was relatively limited, precluding assessment of long-term outcomes. Additionally, residual confounding cannot be entirely excluded, and the lack of prospective randomized evidence limits causal inference. Economic burden and quality-of-life outcomes were not assessed but are important considerations for future research. Finally, because HAIC response is routinely assessed after two treatment cycles and patients with an anticipated survival of less than 3 months are generally not considered candidates for HAIC, the study population may have been enriched for clinically stable patients suitable for planned HAIC-based therapy, potentially introducing selection bias and overestimating absolute OS and PFS despite survival being calculated from first HAIC initiation. Therefore, these findings should be interpreted as hypothesis-generating real-world evidence. Given the retrospective design, potential residual confounding, and the predominantly HBV-related population from experienced HAIC centers, prospective multicenter studies and randomized controlled trials are warranted to confirm the observed survival benefits and establish the generalizability of these results.

In conclusion, this multicenter retrospective study, HTA was associated with improved overall survival compared with HL among patients with unresectable HCC, although no significant differences were observed in PFS, ORR, or DCR. Given the retrospective design, potential residual confounding, and limited generalizability to broader international populations, these findings should be interpreted as preliminary real-world evidence. Prospective studies, preferably randomized trials, are warranted to validate the survival benefit and clarify the optimal treatment strategy in this setting.

## Data Availability

The raw data supporting the conclusions of this article will be made available by the authors, without undue reservation.

## Supplementary material

Supplementary Figure 1 Kaplan–Meier curves for OS comparing patients who underwent HTA versus HL before **(a)** and after **(b)** PSM, following exclusion of patients who underwent conversion resection. *P* values were calculated using Log-rank test. PSM, propensity score matching; HL, HAIC plus lenvatinib; HAIC, hepatic arterial infusion chemotherapy; HTA, HAIC combined with bevacizumab and atezolizumab; OS, overall survival; PFS, progression-free survival; HR: hazard ratio; CI, confidence interval.

Supplementary Table 1 Baseline covariates of the study patients before and after PSM Values are presented as n (%). PSM, propensity score matching; SMD, standardized mean differences; HL, HAIC plus lenvatinib; HAIC, hepatic arterial infusion chemotherapy; HTA, HAIC combined with bevacizumab and atezolizumab; ECOG, Eastern Cooperative Oncology Group; PS, performance score; HBV, hepatitis B virus; HCV, hepatitis C virus; ALBI, albumin-bilirubin; AFP, alpha-fetoprotein; PVTT, portal vein tumor thrombus.
