# Supplementary material for: HAIC combined with atezolizumab and bevacizumab versus lenvatinib in advanced hepatocellular carcinoma: a multicenter propensity score matching analysis
Source: Front Oncol. 2026 Aug 24;16:1880527. doi: 10.3389/fonc.2026.1880527 (PMC13547088; doi:10.3389/fonc.2026.1880527)
Supplement: Supplementary file 1 [file Table1.pdf]

Supplementary table 1 Baseline covariates of the study patients before and after PSM

Characteristics	Before matching			After matching		
	HL (n = 187)	HTA (n = 119)	SMD	HL (n = 106)	HTA (n = 106)	SMD
**Age** (Mean ± SD, year)	52 ± 12	48 ± 12	-0.337	50 ± 12	49 ± 12	-0.076
< 50	76 (40.6%)	60 (50.4%)	0.196	48 (45.3%)	53 (50.0%)	0.094
≥ 50	111 (59.4%)	59 (49.6%)	-0.196	58 (54.7%)	53 (50.0%)	-0.094
**Sex**						
Female	14 (7.5%)	14 (11.8%)	0.133	8 (7.5%)	9 (8.5%)	0.029
Male	173 (92.5%)	105 (88.2%)	-0.133	98 (92.5%)	97 (91.5%)	-0.029
**ECOG-PS**						
0	140 (74.9%)	107 (89.9%)	0.500	90 (84.9%)	94 (88.7%)	0.025
1	47 (25.1%)	12 (10.1%)	-0.500	16 (15.1%)	12 (11.3%)	-0.025
**Etiology**						
No-hepatitis	12 (6.4%)	11 (9.2%)	0.098	6 (5.7%)	6 (5.7%)	0
HBV	174 (93.0%)	107 (89.9%)	-0.104	99 (93.4%)	99 (93.4%)	0
HCV	1 (0.5%)	1 (0.8%)	0.033	1 (0.9%)	1 (0.9%)	0
**Child-Pugh**						
A	165 (88.2%)	105 (88.2%)	0	93 (87.7%)	92 (86.8%)	-0.029
B	22 (11.8%)	14 (11.8%)	0	13 (12.3%)	14 (13.2%)	0.029
**ALBI**						
1	67 (35.8%)	49 (41.2%)	0.109	46 (43.4%)	42 (39.6%)	-0.077
2	116 (62.0%)	68 (57.1%)	-0.099	58 (54.7%)	62 (58.5%)	0.076
3	4 (2.1%)	2 (1.7%)	-0.036	2 (1.9%)	2 (1.9%)	0
**Size** (Mean ± SD, cm)	12.0 ± 3.3	12.5 ± 3.4	0.143	12.3 ± 3.7	12.1 ± 3.2	-0.068
< 10	52 (27.8%)	33 (27.7%)	-0.002	31 (29.2%)	30 (28.3%)	-0.021
≥ 10	135 (72.2%)	86 (72.3%)	0.002	75 (70.8%)	76 (71.7%)	0.021
**Number**						
Single	77 (41.2%)	26 (21.8%)	-0.468	24 (22.6%)	24 (22.6%)	0
Multiple	110 (58.8%)	93 (78.2%)	0.468	82 (77.4%)	82 (77.4%)	0
**AFP** (ug/L)						
< 400	56 (29.9%)	49 (41.2%)	0.228	41 (38.7%)	40 (37.7%)	-0.019
≥ 400	131 (70.1%)	70 (58.8%)	-0.228	65 (61.3%)	66 (62.3%)	0.019
**PVTT**						
Absent	35 (18.7%)	22 (18.5%)	-0.006	18 (17.0%)	17 (16.0%)	-0.024
Present	152 (81.3%)	97 (81.5%)	0.006	88 (83.0%)	89 (84.0%)	0.024
**Extrahepatic metastasis**						
Absent	85 (45.5%)	52 (43.7%)	-0.035	48 (45.3%)	46 (43.4%)	-0.038
Present	102 (54.5%)	67 (56.3%)	0.035	58 (54.7%)	60 (56.6%)	0.038

Values are presented as n (%).

PSM, propensity score matching; SMD, standardized mean differences; HL, HAIC plus lenvatinib; HAIC, hepatic arterial infusion chemotherapy; HTA, HAIC combined with bevacizumab and atezolizumab; ECOG, Eastern Cooperative Oncology Group; PS, performance score; HBV, hepatitis B virus; HCV, hepatitis C virus; ALBI, albumin-bilirubin; AFP, alpha-fetoprotein; PVTT, portal vein tumor thrombus.
